# Disconnection of pulmonary and systemic arterial stiffness in COPD

**DOI:** 10.2147/COPD.S160077

**Published:** 2018-05-28

**Authors:** Jonathan R Weir-McCall, Patrick SK Liu-Shiu-Cheong, Allan D Struthers, Brian J Lipworth, J Graeme Houston

**Affiliations:** 1 Division of Molecular and Clinical Medicine, Medical Research Institute, University of Dundee, Dundee, UK; 2 Scottish Centre for Respiratory Research, Medical Research Institute, University of Dundee, Dundee, UK

**Keywords:** pulmonary vascular, cardiovascular, COPD, arterial compliance, ventricular function

## Abstract

**Background:**

Both pulmonary arterial stiffening and systemic arterial stiffening have been described in COPD. The aim of the current study was to assess pulse wave velocity (PWV) within these two arterial beds to determine whether they are separate or linked processes.

**Materials and methods:**

In total, 58 participants with COPD and 21 healthy volunteers (HVs) underwent cardiac magnetic resonance imaging (MRI) and were tested with a panel of relevant biomarkers. Cardiac MRI was used to quantify ventricular mass, volumes, and pulmonary (pulse wave velocity [pPWV] and systemic pulse wave velocity [sPWV]).

**Results:**

Those with COPD had higher pPWV (COPD: 2.62 vs HV: 1.78 ms^−1^, *p*=0.006), higher right ventricular mass/volume ratio (RVMVR; COPD: 0.29 vs HV: 0.25 g/mL, *p*=0.012), higher left ventricular mass/volume ratio (LVMVR; COPD: 0.78 vs HV: 0.70 g/mL, *p*=0.009), and a trend toward a higher sPWV (COPD: 8.7 vs HV: 7.4 ms^−1^, *p*=0.06). Multiple biomarkers were elevated: interleukin-6 (COPD: 1.38 vs HV: 0.58 pg/mL, *p*=0.02), high-sensitivity C-reactive protein (COPD: 6.42 vs HV: 2.49 mg/L, *p*=0.002), surfactant protein D (COPD: 16.9 vs HV: 9.13 ng/mL, *p*=0.001), N-terminal pro-brain natriuretic peptide (COPD: 603 vs HV: 198 pg/mL, *p*=0.001), and high-sensitivity troponin I (COPD: 2.27 vs HV: 0.92 pg/mL, *p*<0.001). There was a significant relationship between sPWV and LVMVR (*p*=0.01) but not pPWV (*p*=0.97) nor between pPWV and RVMVR (*p*=0.27).

**Conclusion:**

Pulmonary arterial stiffening and systemic arterial stiffening appear to be disconnected and should therefore be considered independent processes in COPD. Further work is warranted to determine whether both these cause an increased morbidity and mortality and whether both can be targeted by similar pharmacological therapy or whether different strategies are required for each.

## Introduction

Despite being a disease of the pulmonary parenchyma, COPD is associated with significant cardiac morbidity and mortality, with up to 43% of mortality in COPD secondary to cardiovascular causes.^1^–^3^ Even in the absence of pulmonary hypertension, those with COPD demonstrate right ventricular (RV) dysfunction,^4^,^5^ and diastolic and systolic left heart failures are present in >20% of those with moderate-to-severe COPD.^6^,^7^

Arteriosclerosis is the stiffening of the arterial wall and is predominantly a result of aging and rising mean arterial pressure in both the systemic and pulmonary circulation.^8^–^11^ An increase in arterial stiffening leads to an increased afterload and an inefficient energy transfer from the ventricles to the vasculature, resulting in an increased cardiac workload.^12^,^13^ Increased stiffening of both the systemic and pulmonary circulation has been described in COPD, independent of traditional risk factors.^14^,^15^ Due to its adverse hemodynamic effects, arterial stiffening is a well-established risk factor for future cardiovascular mortality.^16^ Multiple studies have demonstrated systemic arterial stiffening in those with COPD when compared with matched smokers and for this stiffening to correlate with the percentage of emphysema.^14^,^17^ Several studies have also shown COPD therapy to result in an improvement in arterial stiffness, although this observation has not been consistent across all studies.^18^–^20^ Pulmonary arterial stiffness has also been shown to correlate with exercise capacity and right heart function in COPD.^21^ It has also been shown that those with COPD who are taking statins have lower pulmonary pressures on right heart catheterization compared with those not on statins and that statins are associated with improved functional capacity and lower mortality in COPD.^22^–^24^ Given that statins improve systemic cardiovascular disease and mediate adverse vascular remodeling in atherosclerosis,^25^,^26^ it may thus be posited that the disease process afflicting the pulmonary arteries and aorta may simply be the same pathological process resulting from a COPD-induced accelerated vascular degeneration.

Thus, the aim of the current study was to examine the association between aortic and pulmonary arterial stiffening using pulse wave velocity (PWV) and their effects on cardiac remodeling with the hypotheses that 1) aortic stiffening and pulmonary stiffening occur in parallel; 2) inflammatory markers would explain this interaction; and 3) elevated PWV would be associated with adaptive ventricular remodeling with an increased ventricular mass to end diastolic volume ratio.

## Materials and methods

Between July 2014 and May 2016, participants were recruited from primary and secondary care clinics and from community-based spirometry and research databases. Inclusion criteria for the study were the following: 40–85 years of age with a diagnosis of COPD, based on the current Global Initiative for Chronic Obstructive Lung Disease guidelines of postbronchodilator forced expiratory volume in 1 second/forced vital capacity <0.7 with a history of smoking.^27^ Exclusion criteria were the following: history of cardiac condition, including but not limited to ischemic heart disease, valvular disease (mild functional regurgitation allowed), arrhythmia, cardiomyopathy, congestive cardiac failure, or congenital cardiac disease; previous cardiac or thoracic operation; other coexistent lung condition; connective tissue disease or systemic vasculitis; severe renal impairment (epidermal growth factor receptor <30 mL/min); or contraindication for magnetic resonance imaging (MRI). All recruited participants underwent a screening echocardiogram to exclude significant silent left ventricular systolic dysfunction (ejection fraction <45%).

In total, 104 participants with COPD were screened with 37 excluded due to either coexistent coronary artery disease, atrial fibrillation, prior thoracotomy, coexistent lung condition, left ventricular systolic dysfunction on echo, or no history of smoking. This left 67 participants in the cohort. Of these, 56 participants were included in the final analysis (6 participants were excluded due to incomplete scan secondary to claustrophobia, 1 due to a history of metal fragments in the orbits not picked up in screening, and 4 due to inadequate image quality for analysis). All the participants gave written informed consent for the study that was conducted in accordance with the Declaration of Helsinki and was approved by the East of Scotland Research Ethics Committee 1 (Reference Number: 14/ES/0034).

A healthy control (HC) group was recruited with the age and sex of them approximately matched to the COPD cohort, with no prior history of cardiac or pulmonary pathology.

All COPD participants underwent spirometry, single breath diffusion (diffusing capacity of the lungs for carbon monoxide [DLCO]), a 6-minute walk test (6MWT), and a cardiac MRI on the same day. All HCs underwent a cardiac MRI. Spirometry, DLCO, and 6MWT were performed as per European Respiratory Society/American Thoracic Society guidelines.^28^,^29^ The blood samples of all the participants were taken at the time of their attendance for the MRI scan.

### MRI

Images were acquired on a 32–radiofrequency RF cardiac receiver channel, 3-Tesla MRI scanner (Prisma; Siemens Medical Solutions, Erlangen, Germany). A three-plane localizer was first obtained, following which 4-chamber, 2-chamber, and short-axis localizers of the heart were obtained. An axial half-Fourier acquisition turbo spin echo stack was acquired of the chest.

From these, a balanced steady-state free precession (bSSFP) stack was performed in breath-hold from the atrioventricular ring to the apex. To plan the main pulmonary artery (MPA) phase contrast sequence, a bSSFP sequence of the RV outflow tract was performed following which an orthogonal plane was acquired to optimally visualize the MPA and pulmonary valve. A bSSFP sequence was then performed through the MPA slice and was positioned midway between the valve and the bifurcation of the pulmonary artery in order to avoid both structures throughout the cardiac cycle. A free-breathing phase contrast sequence (slice thickness =6 mm, time to relaxation/time to echo (TR/TE) =12/4 ms, number of averages =1, phases =80, velocity encoding =150 cm/s, bandwidth/pixel =340 Hz, flip angle =15°, field of view =320×320 mm^2^, matrix =512×512) was then performed in the same position as previously described.^30^ For aortic PWV measurement, a two-dimensional gradient echo (fast low-angle shot [FLASH] was first acquired of the aorta in a “candy stick” double-oblique orientation (parameters: TR/TE =40/1.2 ms; flip angle =15°, slice thickness =8 mm, 23 cardiac phases, number of averages =1, a pixel size of 1.5×1.5 mm^2^, bandwidth =475 Hz/pixel). Two-phase contrast sequences were then obtained, the first positioned through the aortic arch at the level of the pulmonary bifurcation and the second slice placed through the proximal abdominal aorta just distal to the aortic hiatus. The same phase contrast sequence was used as for the pulmonary PWV measurement.

### Image analysis

The images were exported with image analysis performed by using CVI 42 (Circle Cardiovascular Imaging Inc., Calgary, Alberta, Canada).

#### Ventricular quantification

Epicardial and endocardial contours were drawn around the right ventricle at end systole and end diastole. Trabeculae were included in the mass measurement and excluded from the volume calculation. The septum was treated as belonging to the left ventricle and was excluded from the RV mass. RV mass and volumes were normalized to height^1.7^.

#### Pulmonary PWV

For the time component, the phase and magnitude images were pulled up side by side. A contour was manually drawn around the perimeter of the vessel, then propagated throughout the cardiac cycle, and corrected as when automatic contouring led to erroneous boundaries. The program then automatically calculated area, flow, and velocity data, which were exported to Excel 2010 (Microsoft Corporation, Redmond, WA, USA). The area and flow were plotted against one another during early systole, which was defined as the time period in systole during which both the vessel area and flow were simultaneously increasing. The PWV was then calculated as described by Davies et al:^31^

$$\text{PWV}=\sqrt{\frac{\sum \Delta Q^{2}}{\sum \Delta A^{2}}}$$

where Q =vessel flow and A=vessel area.

#### Aortic PWV

The distance was measured along the aorta between the two analysis planes (Δx value) using candy stick FLASH and the time delay calculated as the time delay between the arrival of the foot of the pulse wave at the ascending aorta and abdominal aorta. To calculate the latter, the flow curves from the ascending thoracic aorta and the abdominal aorta were plotted, and the time to the systolic upstroke of the waves was then calculated. The arrival time of the flow wave was identified as the intersection between the systolic upstroke and baseline flow. The systolic upstroke was calculated as the line through the data points lying between 20% and 80% of the maximum flow rate.^32^ The baseline was the horizontal line at minimum velocity before systole. Pulse wave was calculated using the following equation:

$$TT\;\text{PWV}=\frac{\Delta d}{\Delta t}$$

where *TT* stands for transit time, *d* stands for distance between the imaging planes, and *t* stands for time of systolic upstroke arrival.

### Biomarkers

In order to better understand the links between COPD, vascular stiffening, and cardiac remodeling, several families of biomarkers were assessed: 1) connective tissue markers – carboxymethyllysine (CML), matrix metalloproteinase-9 (MMP-9), and fibroblast growth factor-23 (FGF-23); 2) inflammatory markers – high-sensitivity C-reactive protein (hsCRP), interleukin-6 (IL-6), and fibrinogen; 3) pulmonary markers – club cell secretory protein-16 (CC-16) and surfactant protein D (SPD); 4) cardiac markers – high-sensitivity troponin I (hsTrop I) and N-terminal pro-brain natriuretic peptide (NT-proBNP); and 5) lipid markers – total cholesterol (TC), high-density lipoprotein (HDL), low-density lipoprotein (LDL), and triglycerides.

For this analysis, blood samples were drawn into 3×4 mL EDTA tubes, which were then centrifuged at 2,000× *g* at room temperature for 15 minutes before the plasma from this was removed and then stored at −80°C. All the samples were stored until the study was completed and then analyzed en bloc. Table S1 presents the details of the manufacturers and interassay (plate-to-plate) and intra-assay coefficients of variations.

### Statistics

Descriptive statistics were used for the analysis of the demographic and clinical features of the cohorts with data expressed as mean ± SD. Normality and equality of variances of the variables were tested. An independent sample *t*-test was used to compare the differences in continuous variables between the HCs and COPD cohort. *χ*^2^, Fisher’s exact, and Mann–Whitney *U* tests were used as appropriate to compare differences in ordinal and nominal data between the groups. Pearson or Spearman rank coefficients were used to assess the correlation between aortic and pulmonary PWV and to look at the correlates of both of these with baseline demographic, spirometric, and MRI factors in the COPD cohort. Multiple linear regression analysis was performed with systemic pulse wave velocity (sPWV) and pulmonary pulse wave velocity (pPWV) entered separately as the dependent variables, with those factors which were (*p*<0.1) in single variable analysis entered as independent variables. All data were analyzed using SPSS package (Version 21.0; IBM Corporation, Armonk, NY, USA). Significance was assumed when *p*<0.05.

## Results

In total, 56 COPD patients (67.4±9.0 years, 55% male) and 20 HCs (60.4±5.1, 48% male) completed the study protocol. Despite approximate age and sex matching, those with COPD were significantly older (*p*<0.001) and had a higher body mass index (BMI; COPD: 26.8±5.3 kg/m^2^ vs HC: 24.6±2.5 kg/m^2^, *p*=0.02). While those with COPD had a significantly higher prevalence of hypertension, there was no significant difference in blood pressure or pulse pressure due to a higher prevalence of antihypertensive medication prescription. Table 1 presents the full baseline characteristics.

Those with COPD demonstrated a significantly higher pulmonary PWV (COPD: 2.6±1.3 ms^−1^ vs HC: 1.8±0.7 ms^−1^, *p*=0.006) and a trend toward a higher aortic PWV (COPD: 8.7±2.7 ms^−1^ vs HC: 7.4±2.1 ms^−1^, *p*=0.06). Compared with the HCs, those with COPD had a significantly lower RV stroke volume (COPD: 31.7±7.0 mL/m^1.7^ vs HC: 36.6±5.9 mL/m^1.7^, *p*=0.007) and a higher RV mass/volume ratio (RVMVR; COPD: 0.29±0.05 g/mL vs HC: 0.25±0.04 g/mL, *p*=0.012). Those with COPD also had a significantly higher left ventricular mass/volume ratio (LVMVR; COPD 0.78±0.13 g/mL vs HC: 0.70±0.09 g/mL, *p*=0.009; see Table 2 for full ventricular and pulmonary arterial parameters).

In those with COPD, pulmonary PWV was correlated with percentage predicted total lung capacity (ρ=0.28, *p*=0.046), inversely correlated with BMI (ρ=−0.28, *p*=0.04), and correlated with increasing diastolic blood pressure (ρ=0.36, *p*=0.01) with a trend toward a positive correlation with percentage predicted residual lung volume (ρ=0.25, *p*=0.08; see Table S2 for full correlates). Applying a conservative Bonferroni correction for multiple comparisons would result in a significance level of *p*<0.0008, with none of the variables meeting this level. On multiple-variable backward linear regression, diastolic blood pressure (ß=0.28, *p*=0.035) and percentage predicted total lung capacity (ß=0.30, *p*=0.028) remained significantly associated with pPWV.

In comparison, aortic PWV showed a significant association with age (ρ=0.47, *p*<0.001), systolic blood pressure (ρ=0.32, *p*=0.02), and percentage predicted transfer coefficient for carbon monoxide (KCO) (ρ=0.43, *p*=0.001) and a trend toward a significant association with percentage predicted DLCO (ρ=0.27, *p*=0.050) and pulse pressure (ρ=0.25 *p*=0.067). Applying a conservative Bonferroni correction for multiple comparisons would result in a significance level of *p*<0.0008, as a result of which only age would remain a significant correlate of sPWV. On linear regression, age (ß=0.30, *p*=0.02), systolic blood pressure (ß=0.27, *p*=0.04), and percentage predicted KCO (ß=0.37, *p*=0.003) all remained significantly associated with aortic PWV.

**Figure 1:**
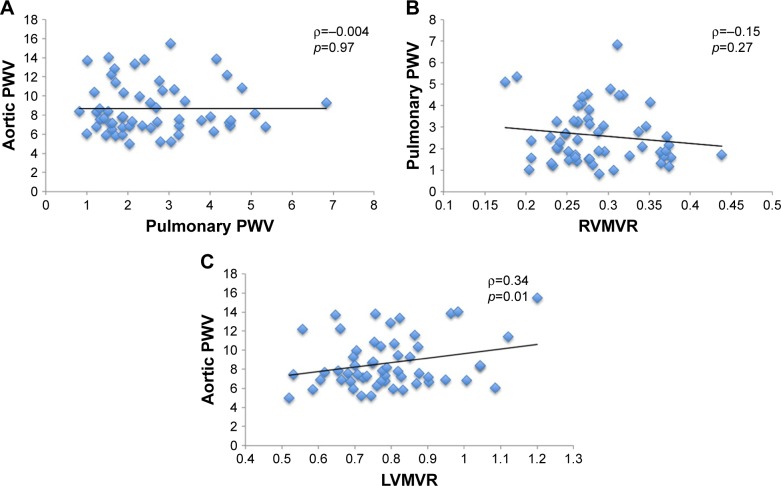
Scatterplots of (**A**) aortic PWV against pulmonary PWV; (**B**) pulmonary PWV against RVMVR; and (**C**) aortic PWV against LVMVR. **Abbreviations:** LVMVR, left ventricular mass/volume ratio; PWV, pulse wave velocity; RVMVR, right ventricular mass/volume ratio.

sPWV was significantly associated with LVMVR (ρ=0.34, *p*=0.01), while no such correlation was found between pPWV and RVMVR (ρ=−0.15, *p*=0.27). Similarly, there was no significant association between aortic and pulmonary PWV (ρ=−0.004, *p*=0.97; Figure 1).

Those with COPD showed significantly higher levels of the following: inflammatory markers IL-6 (COPD: 1.38±1.38 pg/mL vs healthy volunteers [HVs]: 0.58±0.66 pg/mL, *p*=0.018) and hsCRP (COPD: 6.42±7.68 mg/L vs HV: 2.49±2.84 mg/L, *p*=0.002); connective tissue markers MMP-9 (COPD: 121±72 ng/mL vs HV: 50±19 ng/mL, *p*<0.001); lung tissue marker SPD (COPD: 16.9±12.7 ng/mL vs HV: 9.13±5.41 ng/mL, *p*=0.001); and cardiac markers NT-proBNP (COPD: 603±839 pg/mL vs HV: 198±165 pg/mL, *p*=0.001) and hsTrop I (COPD: 2.27±1.90 pg/mL vs HV: 0.92±0.49 pg/mL, *p*<0.001).

No significant difference was found in FGF-23, CC-16, CML, fibrinogen, cholesterol, HDL, or LDL (*p*>0.05 for all).

In those with COPD, there was a weak correlation between pPWV and hsCRP (ρ=−0.28, *p*=0.04), TC (ρ=0.28, *p*=0.044), and NT-proBNP (ρ=0.28, *p*=0.048). However, on linear regression accounting for diastolic blood pressure, the significance of these was lost. For sPWV, there was a weak correlation with CC-16 (ρ=0.31, *p*=0.028), TC (ρ=0.33, *p*=0.019), and LDL (ρ=0.32, *p*=0.023). However, on linear regression accounting for age and systolic blood pressure, these associations were no longer significant.

## Discussion

In this study, we have seen that 1) both pulmonary and systemic arteries are stiffer in COPD compared with controls; 2) aortic PWV and pulmonary PWV are associated with different clinical parameters and biomarkers from one another; and 3) aortic but not pulmonary PWV is associated with ventricular hypertrophy.

Our finding of increased aortic stiffness is consistent with previous work demonstrating increased aortic PWV in those with COPD.^33^ In comparison with the previous studies that used carotid femoral PWV, the current study used MRI for the assessment of central aortic PWV. The two techniques have been shown to correlate well with one another, although carotid–femoral PWV significantly overestimates central aortic PWV due to the inclusion of the stiffer peripheral vessels in its calculation and inaccuracies in path length estimation.^34^–^36^ In addition, it excludes the ascending aorta from its measurement, with this playing a greater role in left ventricular remodeling compared with the rest of the aorta.^37^ By its direct assessment of central aortic PWV, the current study provides valuable insights into the interaction between central aortic stiffness and left ventricular remodeling. The underlying cause of the increased systemic arterial stiffness in COPD is still a topic of debate. While a previous association between aortic PWV and inflammatory markers has been described,^38^ other groups have found no such correlation.^39^,^40^ In the current study, we found that in those with COPD the aortic PWV was associated with age and blood pressure, but not with inflammatory markers. Our finding of a significant association between LVMVR – which is the earliest measure of concentric myocardial hypertrophic remodeling^41^ – and aortic PWV provides significant mechanistic insight into the interface between the pulmonary and cardiovascular disease evident in COPD. As with increased aortic PWV, multiple studies have documented left ventricular hypertrophy in COPD, demonstrating both a high prevalence and significant implications for mortality in this group.^42^–^44^ While the association between PWV and left ventricular mass has been described in the general population,^45^,^46^ to the best of the authors’ knowledge, no studies have examined the role of the aortic arterial stiffness as a linking mechanism in COPD. Indeed, a previous study ascribed the observed left ventricular hypertrophy to pulmonary hyperinflation, but did not measure or account for aortic stiffness.^43^ Given that LVMVR is a known risk factor for future cardiovascular events, this association may help explain both the increased cardiovascular risk in COPD and the beneficial effects in COPD of β-blockers – a known regressor of left ventricular mass.^47^–^49^

Increased pulmonary arterial stiffness in COPD has been previously reported both invasively on right heart catheterization and using pulsatility on MRI.^4^,^15^ Invasive pulmonary PWV has been demonstrated to correlate well with invasive pressure measurements in those with pulmonary hypertension.^50^ Pulmonary PWV as measured on MRI provides the benefit of not requiring an invasive procedure to obtain direct information on the arterial wall. It also provides additional information over pulmonary pulsatility as this latter metric is a flow-dependent measure of the relative change in the area of the pulmonary artery throughout the cardiac cycle. Thus, assuming a steady pulmonary arterial wall stiffness, a fall in stroke volume will result in a fall in pulmonary pulsatility. As both a reduced stroke volume and a reduced pulmonary pulsatility have been described in COPD,^15^,^51^ pulmonary PWV is a useful measure to better interrogate pulmonary arterial remodeling.

Despite those with COPD having significantly elevated hsCRP, IL-6, MMP-9, NT-proBNP, and hsTrop I, none of these markers showed any significant correlation with either aortic or pulmonary PWV. In the current study, we found no significant link between aortic and pulmonary PWV, with no overlap of clinical correlates or biomarkers. Thus, it would appear that the pulmonary vascular remodeling in COPD occurs separately to systemic arterial stiffening. Previous work has described pulmonary arterial stiffening to be significantly associated with exercise-induced elevations in pulmonary pressures;^21^,^52^ however, we found no link between this and RV remodeling; therefore, while the pressures may elevate in exercise, the clinical significance of this is uncertain. Longitudinal studies will be required to determine the long-term implications of pulmonary arterial stiffening.

Given the lack of significant correlation between pulmonic and arterial stiffening and biomarkers, particularly the lack of association with inflammation, another link between COPD and arterial stiffening must also be considered. It has been previously demonstrated that the two strongest predictors of aortic PWV are age and blood pressure.^8^ The former of these reflects a gross marker of the total pulsatile cycles the aorta has undergone, while the latter reflects the pressure at which the walls have been distended during these cycles. Thus, COPD, with its higher heart rate secondary to lower stroke volumes as found in this and other studies,^51^,^53^ may accelerate arterial stiffening without the need for an inflammatory link. A study in a population free from cardiovascular disease has shown an elevated resting heart rate to be associated with increased aortic stiffness.^54^ In those with COPD, resting heart rate is associated with cardiovascular mortality, but a direct link between heart rate and arterial stiffness in COPD has yet to be evaluated.^53^,^55^ While elevated heart rate would affect the aortic and pulmonary circulation in a similar pattern, the underlying distending pressures in the two systems vary independently of one another; thus the two circulations may remodel at a different rate even with the same accelerating factor. Another alternative is the role of autonomic nervous system activation, which has been described in COPD and is known to be associated with PWV.^56^–^58^

There are some limitations to the current study. Patients with known coronary artery disease were excluded; thus, the results may not be representative of the wider COPD cohort in whom cardiovascular disease is common. Despite attempted matching, the controls were younger than those with COPD and more likely to be on treatment for hypertension although the blood pressures were well matched. As age is a significant contributor to PWV, this may have contributed to the difference between the two groups although our observations are concordant with the current literature.^8^ Unfortunately, due to the limited group sizes, particularly of the controls, the data did not meet assumptions required for the analysis of covariance; therefore, multiple variable modeling to account for these could not be performed. We also used two different techniques for the MRI assessment of the aortic and pulmonary PWV, using a transit time method in the aorta and a flow area technique within the pulmonary artery. This was done as the former is more accurate in longer vessels such as the aorta;^32^ however, the short pulmonary artery is less amenable to this due to the rapid transit times, and prior work has shown the transit time within the pulmonary arteries to be less reproducible compared with the flow area technique.^30^ In addition, the two techniques demonstrate a reasonable agreement with one another minimizing the potential significance of this.^59^ Finally, if we had enrolled more severe hypoxic COPD patients in GOLD stages III/IV (ie, FEV<50%), then perhaps we would have seen more pronounced RV remodeling.

## Conclusion

In conclusion, both aortic and pulmonary arterial stiffening occurs in COPD, but these processes are independent of one another with aortic but not pulmonary PWV, demonstrating a significant association with remodeling in the ventricle. Further work is warranted to determine whether this stiffening causes an increased morbidity and mortality and whether both can be targeted by similar pharmacological therapy or whether different strategies are required for each.

## Supplementary materials

**Table S1:** Intra-assay and interassay CoV for the biomarkers

Variable	Manufacturer details	Interassay (plate-to-plate CoV, %)	Intra-assay (CoV, %)
Surfactant protein D	Bio-Techne, Minneapolis, MN, USA	9.1	4.0
Club cell protein-16	Cloud-Clone Corp., Katy, TX, USA	5.4	10.4
Fibroblast growth factor-23	Cloud-Clone Corp.	9.6	6.2
hsCRP	Kalon Biological Ltd., Guildford, UK	5.3	3.8
hsTrop I	Quanterix, Lexington, MA, USA	8.5	7.7
NT-proBNP	Meso Scale Discovery, Rockville, MD, USA	10.5	5.2
Fibrinogen	Cusabio, Houston, TX, USA	4.1	3.1
IL-6	Meso Scale Discovery	4.2	4.8
MMP-9	Bio-Techne	8.3	5.6
Carboxymethyllysine	Cusabio	10.2	5.5

**Abbreviations:** CoV, coefficient of variation; hsCRP, high-sensitivity C-reactive protein; hsTrop I, high-sensitivity troponin I; IL-6, interleukin-6; MMP-9, metalloproteinase-9; NT-proBNP, N-terminal pro-brain natriuretic peptide.

**Table S2:** Correlation coefficients between aortic PWV and pulmonary PWV and demographic and ventricular measures

Variable	Pulmonary PWV		Aortic PWV	
	ρ	*p*-value	ρ	*p*-value
Age	0.03	0.81	0.45	<0.001
BMI (kg/m ^2^)	−0.28	0.036	−0.04	0.78
Heart rate (bpm)	0.21	0.15	0.02	0.91
Systolic BP (mmHg)	0.15	0.29	0.31	0.03
Diastolic BP (mmHg)	0.36	0.01	0.22	0.12
Pulse pressure (mmHg)	0.000	1	0.24	0.09
SpO _2_ (%)	−0.06	0.68	−0.07	0.64
Pack-years	0.004	0.98	−0.13	0.36
FEV _1_, % predicted	−0.15	0.28	0.05	0.70
FVC, % predicted	−0.06	0.64	−0.03	0.85
FEV _1_/FVC	−0.17	0.21	0.07	0.64
KCO, % predicted	−0.08	0.56	0.46	0.001
RLV, % predicted	0.25	0.08	−0.10	0.50
TLC, % predicted	0.28	0.046	−0.05	0.71
VC, % predicted	−0.02	0.90	0.03	0.83
RLV/TLC	0.20	0.15	0.12	0.40
CML	−0.15	0.29	0.15	0.31
MMP-9	0.10	0.47	0.09	0.55
FGF-23	0.14	0.33	0.06	0.69
CC-16	−0.18	0.21	0.31	0.028
SPD	−0.26	0.07	0.05	0.72
hsCRP	−0.28	0.04	−0.007	0.96
IL-6	−0.26	0.07	−0.02	0.92
Fibrinogen	0.001	0.99	0.16	0.26
TC	0.28	0.044	0.33	0.019
HDL	−0.12	0.40	0.06	0.70
LDL	0.15	0.28	0.32	0.023
Triglycerides	−0.09	0.53	0.12	0.39
hsTrop I	−0.15	0.29	0.17	0.23

**Abbreviations:** BMI, body mass index; BP, blood pressure; CC-16, club cell secretory protein-16; CML, carboxymethyllysine; FEV_1_, forced expiratory volume in 1 second; FGF-23, fibroblast growth factor-23; FVC, forced vital capacity; HDL, high-density lipoprotein; hsCRP, high-sensitivity C-reactive protein; hsTrop I, high-sensitivity troponin I; IL-6, interleukin-6; LDL, low-density lipoprotein; MMP-9, metalloproteinase-9; NT-proBNP, N-terminal pro-brain natriuretic peptide; PWV, pulse wave velocity; RLV, residual lung volume; SPD, surfactant protein D; TC, total cholesterol; TLC, total lung capacity; VA, alveolar volume.

## Figures and Tables

**Table 1:** Demographics of the COPD and healthy control cohorts

Demographics	Healthy controls	COPD cohort	*p*-value
n	20	56	
Age (years)	60.1±4.9	67.5±9.3	<0.001
Sex (male)	9 (45%)	29 (52%)	0.60
BMI (kg/m^2^)	24.6±2.5	26.8±5.3	0.02
Heart rate (bpm)	64.4±12.1	74.0±20.7	0.061
Systolic BP (mmHg)	128.5±13.7	130.8±20.4	0.65
Diastolic BP (mmHg)	75.0±7.9	75.1±8.6	0.95
Pulse pressure (mmHg)	53.5±9.4	55.7±18.7	0.63
SpO_2_	98.4±1.3	96.3±2.5	0.001
Hypertension	2 (10%)	24 (43%)	0.012
Hypercholesterolemia	3 (15%)	3 (5%)	0.18
Diabetes	0 (0%)	9 (16%)	0.1
Smoking status			
Current smoker	2 (10%)	17 (30%)	0.08
Ex-smoker	7 (35%)	39 (70%)	0.009
Never smoker	11 (55%)	0 (0%)	<0.001
Pack-years	4.92±7.5	48.5±23.6	<0.001
Medications			
Aspirin	1 (5%)	9 (16%)	0.28
β-blocker	1 (5%)	3 (5%)	1
Diuretic	0 (0%)	9 (16%)	0.10
Calcium channel blocker	1 (5%)	15 (27%)	0.055
ACEi/ARB	0 (0%)	8 (14%)	0.10
Statin	4 (20%)	14 (25%)	0.77
GOLD (FEV_1_) status			
I		11 (20%)	
II		31 (55%)	
III		13 (23%)	
IV		1 (2%)	
mMRC grade			
0		5 (9%)	
1		26 (46%)	
2		13 (23%)	
3		10 (18%)	
4		2 (4%)	

**Abbreviations:** ACEi, angiotensin converting enzyme inhibitor; ARB, angiotensin receptor blocker; BMI, body mass index; BP, blood pressure; FEV_1_, forced expiratory volume in 1 second; GOLD, Global Initiative for Chronic Obstructive Lung Disease; mMRC, modified Medical Research Council.

**Table 2:** Ventricular quantification, measures of PWV, and bio-markers in the healthy control and COPD cohort

	Healthy controls	COPD cohort	*p*-value
n	20	56	
Right ventricle			
RVEDV (mL/m^1.7^)	58.7±12.1	53.6±11.2	0.09
RVESV (mL/m^1.7^)	22.2±8.2	21.9±7.4	0.87
RVSV (mL/m^1.7^)	36.6±5.9	31.7±7.0	0.007
RVEF (%)	63.3±7.9	59.6±7.9	0.08
RVM (g/m^1.7^)	14.7±2.4	15.2±3.2	0.50
RVMVR (g/mL)	0.25±0.04	0.29±0.05	0.012
Left ventricle			
LVEDV (mL/m^1.7^)	59.0±9.2	56.1±12.1	0.33
LVESV (mL/m^1.7^)	23.4±6.1	23.4±9.4	0.99
LVSV (mL/m^1.7^)	35.6±4.8	32.7±6.7	0.09
LVEF (%)	60.7±6.0	59.1±8.6	0.46
LVM (g/m^1.7^)	41.0±7.4	43.3±9.5	0.33
LVMVR (g/mL)	0.70±0.09	0.78±0.13	0.009
Pulse wave velocity			
Aortic PWV (m/s)	7.35±2.1	8.67±2.7	0.06
Pulmonary PWV (m/s)	1.76±0.7	2.63±1.3	0.006
Biomarkers			
CML (pg/mL)	210±84	260±191	0.27
MMP-9 (ng/mL)	50.2±18.9	121.1±72.2	<0.001
FGF-23 (pg/mL)	26.2±18.2	22.9±6.8	0.27
CC-16 (ng/mL)	24.6±8.5	19.8±10.3	0.07
SPD (ng/mL)	9.12±5.41	16.90±12.71	0.001
hsCRP (mg/L)	2.49±2.84	6.41±7.68	0.002
IL-6 (pg/mL)	0.58±0.66	1.38±1.38	0.018
Fibrinogen (mg/mL)	4.01±1.09	3.89±1.52	0.73
TC (mmol/L)	5.92±0.92	5.41±1.18	0.09
HDL (mmol/L)	1.38±0.58	1.52±0.60	0.39
LDL (mmol/L)	2.93±1.21	2.58±1.44	0.35
Triglycerides (mmol/L)	1.86±0.99	1.60±0.96	0.31
hsTrop I (pg/mL)	0.92±0.49	2.27±1.90	<0.001
NT-proBNP (pg/mL)	198±165	602±839	0.001

**Abbreviations:** CC-16, club cell secretory protein-16; CML, carboxymethyllysine; FGF-23, fibroblast growth factor-23; HDL, high-density lipoprotein; hsCRP, high-sensitivity C-reactive protein; hsTrop I, high-sensitivity troponin I; IL-6, interleukin-6; LDL, low-density lipoprotein; LVEDV, left ventricular end diastolic volume; LVEF, left ventricular ejection fraction; LVESV, left ventricular end systolic volume; LVM, left ventricular mass; LVMVR, left ventricular mass/volume ratio; LVSV, left ventricular stroke volume; MMP-9, metalloproteinase-9; NT-proBNP, N-terminal pro-brain natriuretic peptide; PWV, pulse wave velocity; RVEDV, right ventricular end diastolic volume; RVEF, right ventricular ejection fraction; RVESV, right ventricular end systolic volume; RVM, right ventricular mass; RVMVR, right ventricular mass/volume ratio; RVSV, right ventricular stroke volume; SPD, surfactant protein D; TC, total cholesterol.
